# Scale-Free Brain Quartet: Artistic Filtering of Multi-Channel Brainwave Music

**DOI:** 10.1371/journal.pone.0064046

**Published:** 2013-05-22

**Authors:** Dan Wu, Chaoyi Li, Dezhong Yao

**Affiliations:** 1 Key Laboratory for NeuroInformation of Ministry of Education, School of Life Science and Technology, University of Electronic Science and Technology of China, Chengdu, China; 2 Center for Life Sciences, Shanghai Institutes for Biological Sciences, Chinese Academy of Sciences, Shanghai, China; UC Davis School of Medicine, United States of America

## Abstract

To listen to the brain activities as a piece of music, we proposed the scale-free brainwave music (SFBM) technology, which translated scalp EEGs into music notes according to the power law of both EEG and music. In the present study, the methodology was extended for deriving a quartet from multi-channel EEGs with artistic beat and tonality filtering. EEG data from multiple electrodes were first translated into MIDI sequences by SFBM, respectively. Then, these sequences were processed by a beat filter which adjusted the duration of notes in terms of the characteristic frequency. And the sequences were further filtered from atonal to tonal according to a key defined by the analysis of the original music pieces. Resting EEGs with eyes closed and open of 40 subjects were utilized for music generation. The results revealed that the scale-free exponents of the music before and after filtering were different: the filtered music showed larger variety between the eyes-closed (EC) and eyes-open (EO) conditions, and the pitch scale exponents of the filtered music were closer to 1 and thus it was more approximate to the classical music. Furthermore, the tempo of the filtered music with eyes closed was significantly slower than that with eyes open. With the original materials obtained from multi-channel EEGs, and a little creative filtering following the composition process of a potential artist, the resulted brainwave quartet opened a new window to look into the brain in an audible musical way. In fact, as the artistic beat and tonal filters were derived from the brainwaves, the filtered music maintained the essential properties of the brain activities in a more musical style. It might harmonically distinguish the different states of the brain activities, and therefore it provided a method to analyze EEGs from a relaxed audio perspective.

## Introduction

Music composition originates from the imitation of natural sounds. Some kinds of music came from the nature or the environment. For example, the works of Bandari utilized many natural sounds. In the work *4′33″* of John Cage, all the audible elements during that time were considered as music. Besides the direct reflection of the nature, some music works represented the nature in an abstract way, such as *The Four Seasons* of Vivaldi, *Pastorale Symphony* of Beethoven, and so on. Therefore, music is based on the understanding and abstraction of nature, and it implies the properties of nature and aspiration of aesthetics.

As a product of evolution, the human brain may have some intrinsic feelings of natural aesthetics in the structures and functions, including sounds and music. During the evolution, music originated and developed along with the interactions between human and natural sounds. So the brain activities and signals would be of musical characters potentially constructed by such interactions to a certain extent. The core of brainwave music is to represent the musical attributes of the brain in a musical way.

The study of brain music can be dated from the time people tried to “hear” the hidden brain activity from a noninvasive scalp EEG. The earliest attempt to translate brainwaves into music was made in 1934 [1]. A concert named “Music for Solo Performer” was later presented in 1965 [2], and other similar music pieces followed. In the 1990s, several music generating rules were created from digital filtering or coherent analysis of EEGs [3]. However, in these early works, the mapping rules were rather direct and arbitrary.

In the past ten-odd years, various new strategies of converting EEGs into audible sounds have been proposed and many artificial sound synthesizers have been used for display [4]. And these works can be divided into two main categories of brainwave music systems according to the hierarchy of the features extracted for music generation: the “EEG sonification” and the “Brian-Computer Music Interface”. “EEG sonification” means to translate a few parameters of EEG into the characteristic parameters of music [2], [4], [5], or to use specific events as triggers for the beginning of music tones or other sound events. Usually, the transformation is based on subjectively-defined translation rules [4], [6]. The second category is the musical application of Brain-Computer Interface (BCI) [6]–[8], where induced EEG changes are used to trigger pre-defined music events.

However, all the translations of brain music followed some subjective rules on the basis of the extrinsic characters of EEG, except the scale-free brainwave music (SFBM) technology we proposed, which was based on the intrinsic scale-free properties of both EEG and music [5], [9], reflecting the interior relations between brain signals and music.

Note that our scale-free music pieces were originally atonal. However, the classical or say the traditional music works are usually tonal, and the tonality of music implies the pitch hierarchy. Music composition is usually an imitation of the nature, by which all the information from the environment is abstracted and promoted. Therefore, composition is like filtering in signal processing which removes non-signal parts and maintains useful information. Each composer artificially utilizes an artistic filter to obtain the music according to his understanding and feelings of the nature. Apparently, such artistic filter may be a very important aspect of a composer’s music style. Especially in a quartet, the composer needs to arrange the different parts of the melody, where the artistic filter is used to ensure the consonance of multiple voices.

In the current study, we hypothesized that the cooperation of different brain regions is just the same as the cooperation of voices in a quartet, a very common multi-voice musical style. This hypothesis could help exert the musical potential of brain signals in a macroscopic. To be more specific, how can we realize the brain quartet with multi-channel signals? We suggest using artistic filters designed from the brain signals themselves. Here, we introduced the method of the artistic filter design, and then real EEG signals were adopted to generate the quartet. The music pieces were analyzed and discussed.

## Materials and Methods

### 1. Ethics Statement

All the experiments were conducted according to the principles expressed in the Declaration of Helsinki, and were approved by the Institutional Review Board of University of Electronic Science and Technology of China. The subjects provided written informed consents for the collection of samples and subsequent analyses.

### 2. Data Acquisition and Pre-processing

**Figure 1:**
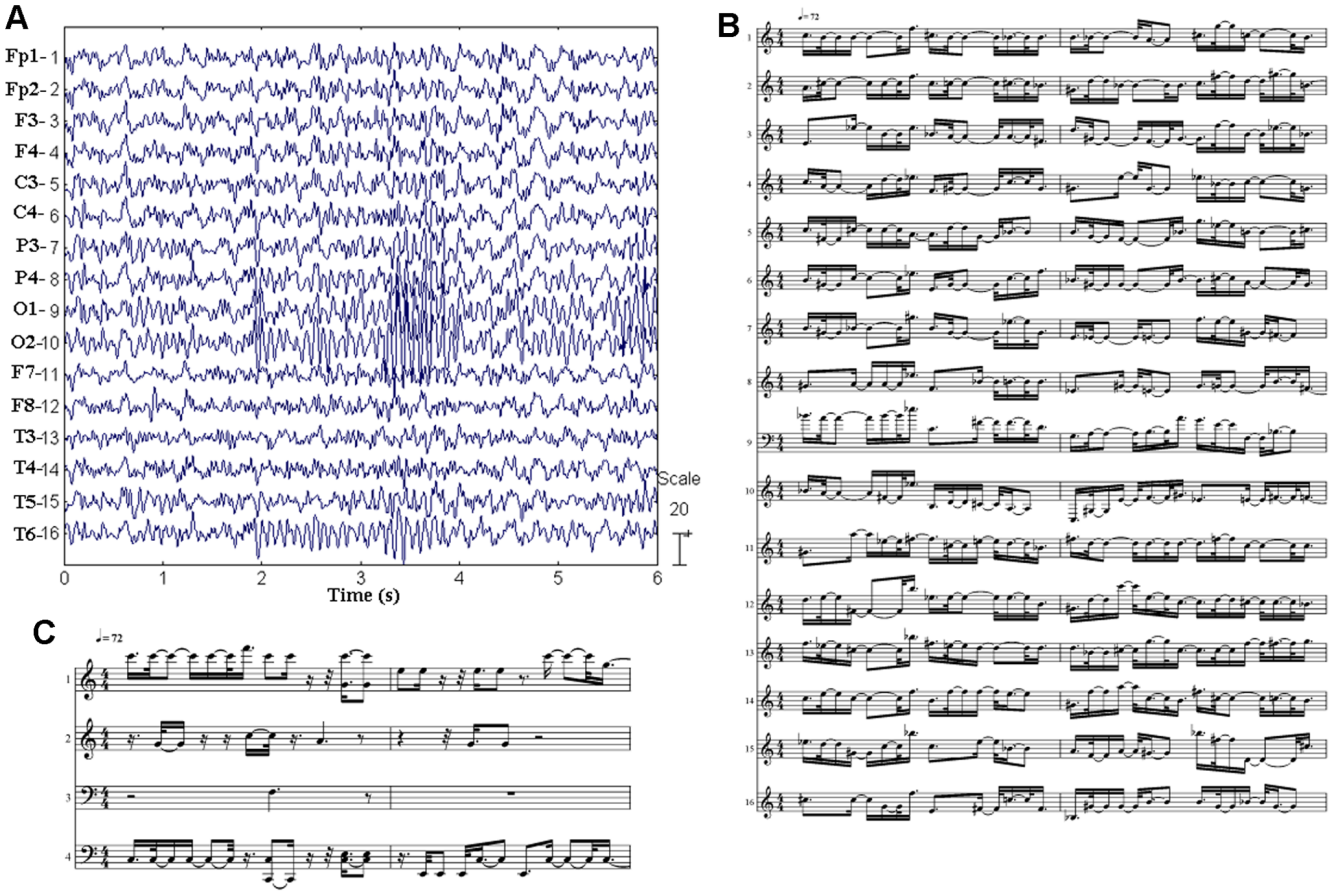
The generation flow of the brain music. The original EEG from 16 electrodes were collected (showed in A) and pre-processed. Then the signals were translated to 16 channels music according to the scale-free brainwave music (SFBM) technology, and the beat filtering was utilized (showed in B). Finally the 16 channels were changed to 4 channels by the tonality filtering (showed in C).

For the resting EEG data acquisition, 40 subjects (age from 23 to 27 years, 20 women, 20 men) were recruited. They were all right-handed and physically and mentally healthy. EEGs were recorded by a 16-channel EEG system with a sampling rate of 1000 Hz and were band-pass filtered from 0.5 Hz to 35 Hz. The subjects were asked to seat on a comfortable chair and keep quiet; EEG data were recorded for 3 minutes with eyes closed and 3 minutes with eyes open. Figure 1A showed the original EEG signals.

After recording, ordinary EEG pre-processing (i.e., artifact rejection, band-pass filtering) was done. The data lasting 60 seconds with no obvious artifact for resting EEG were chosen for music generation. The data were re-referenced to infinity with the REST software [10], [11].

### 3. EEG-music Translation

The selected EEG data were translated in to music sequences per electrode first. A musical note has four essential parameters: timbre, duration, pitch, and intensity. In this study, the timbre of piano was used (other instruments were also acceptable), and the duration, pitch and intensity of a note were obtained respectively from an EEG event period, the wave amplitude and the change of energy.

In the proposed method, for each channel signal, an EEG “event” began when the wave crossed the zero line from negative to positive; and ended at the third crossing. Thus, the duration of an EEG event modulated the duration of a note.

The EEG amplitude was translated into pitch. The pitch of a note is the logarithm of the fluctuation frequency of the instrument. In MIDI information, the value of pitch was an integer from 1 to 127. Here we defined the mapping rule from EEG amplitude (Amp) to musical pitch according to the scale-free rule of both EEG and music. The relation was defined as follows:
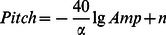
(1)


Here 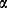 is the scaling exponent of the EEG data, and *n* is a constant. The details for the derivation of equation (1) were presented in the paper [5]. The exponent 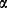 was set around 1 in general. Obviously, the range of Pitch in equation (1) was decided by the Amp, and it was rounding off into an integer.

The EEG power of alpha bands was used to determine the music intensity. Here the music intensity (MI) was assumed to be proportional to the logarithm of the change rate of the average power (AP) according to Fechner’s law [12]. The equation was: 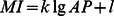. Here MI was an integer ranged from 1 to 127. Such a definition is based on the psychological fact that stimulus information may not be efficiently conveyed by a habitual signal but by a change. This definition was based on the psychological fact that stimulus information may be efficiently conveyed not by a habitual signal but by the change. Meanwhile, to insure smooth and gentle change of intensity [9], the power of alpha bands was selected to determine the music intensity.

### 4. Beat Filtering

Beat filtering was used for adjusting the note duration. In music, the lasting time of notes is represented by beats; for example, a note may last for 2 beats, 1 beat or 1/2 beat etc. So the relations of the different note durations are usually like this: a whole note equals to four beats in 4/4 time, and a half note last two beats, a quarter note is one beats, and so on. There are simple integer ratios for the length of different notes. Generally speaking, the note durations in music works are in accord with these principles, especially in the compositions with obvious rhythms, such as march and minuet. Such a normative rule might be abstracted from the irregular natural voices by composers in the history; thus we also needed to design a beat filter to make the melody of brain activities more musical and cultural.

In our generated music, the shortest note, treated as a demisemiquaver, was defined as a base duration (BD). It was set in each music piece. Therefore the duration of each note in the piece was multiple of BD. The BD was corresponding to a characteristic frequency defined as follows.

The power spectra were analyzed to obtain the four parameters: the max value of the power in alpha band (8–13 Hz) (P_α_), the frequency of the peak (F_α_), the max value of the power in beta band (13.5–35 Hz) (P_β_), and the frequency of peak (F_β_). We defined an empirical threshold (T) of P_α_/P_β_. When P_α_/P_β_>T, BD = 1/F_α_, and when P_α_/P_β_<T, BD = 1/F_β_. During the beat filtering, the duration of each note was rounded to the nearest multiple of BD. Relations between P_α_/P_β_and T were used to distinguish the two eye conditions. Generally, if P_α_/P_β_>T, brain signals were obtained in the EC condition; if P_α_/P_β_<T, the EO condition.

The tempo of music (TM) was also defined and represented by the beats in a minute. If BD was used as a demisemiquaver, the total beats in a minute would be 60/(BD*8), which meant,when P_α_/P_β_>T,TM = 7.5F_α_, and when P_α_/P_β_<T, TM = 7.5F_β_. Figure 1B showed an example of music after beat filtering.

### 5. Tonality Filtering

The first step for tonality filtering was to define the key, which consisted of a main note and mode (Major/Minor). In this study, a statistic method was used for finding the main note. All the notes were put into the 12 pitch classes, and the total duration of every pitch class was counted. The pitch class with largest duration was chosen as the main note. For example, if in a music piece, the lasting time of the pitch class C, including the pitch number 48, 60, 72 etc., in MIDI, were the longest, C would be the main note.

The mode of music is related to emotion or mood. A major music is usually perceived as emotionally positive while a minor is identified to be soft and mellow [13]. In this work, we defined an empirical threshold that when the spectra power of alpha band is lower than the threshold, we take the Major; otherwise the Minor.

The second step was ranking the note stability. In a supposed key, the stability of notes can be measured. Table 1 showed the stability rank of note in both Major and Minor. The most stable note is the “main note”, and the next is “dominant” in all the 24 keys. And for a note in a defined key, the stability is related to its pitch interval from the main note.

**Table 1:** The pitch stability of notes in Major and Minor.

Interval from the mainnote/semitone	Rank in Major	Rank in Minor
0	1	1
1	8	8
2	6	6
3	8	3
4	3	8
5	4	4
6	8	8
7	2	2
8	8	5
9	5	8
10	8	7
11	7	8

In a moment, there were several notes sounding simultaneously, and each of them had a stability rank. The most stable note was extracted at first, then the second stable note, and so on. Four notes were remained at most in this study. Considering that same notes might be obtained from different channels, the notes surviving the filtering were allowed to be repeated only once. For example, if signals from two channels generated notes all ranked the first, only two of them would be remained, and the other must be deleted. After the filtering, the multi-channel sequences were changed into four-channel sequences.

In music, a quartet is a method of instrumentation or vocal by 4 different sounds or voices to make a melodious music or song. In this study, the extracted notes were arranged into four voices. The four-channel sequences were shifted with octaves and consequently put into different pitch ranges. Figure 1C showed an example of music after beat and tonality filtering.

### 6. Evaluation Test

In order to compare the differences between the single channel and the multi-channel brain music quartet, 22 healthy volunteers (average age: 23.2±0.93 years, 6 women, 16 men) participated in this test. None of them reported any neurological disorder, psychiatric disease, or were on medication. All had normal hearing. All of them had never received special musical education.

Volunteers were given four music pieces in the test, the single channel (O1) music with eyes closed and eyes open, the multi-channel quartet under the two conditions. Each music piece lasted 60 seconds. The volunteers were asked to focus on the differences of the music pieces. After listening to the music, they were required to rate for several music parameters on a 9-point scale from 1 to 9 for tempo(1 = very slow and 9 = very fast), valence (1 = very negative and 9 = very positive), arousal(1 = very passive and 9 = very excited), rhythm (1 = less rhythmical and 9 = more rhythmical), musicality (1 = unmusical and 9 = good and pleasing), and richness (1 = very monotonous and 9 = very expressive).

## Results

### 1. The Brain Quartet of the Resting States

**Figure 2:**
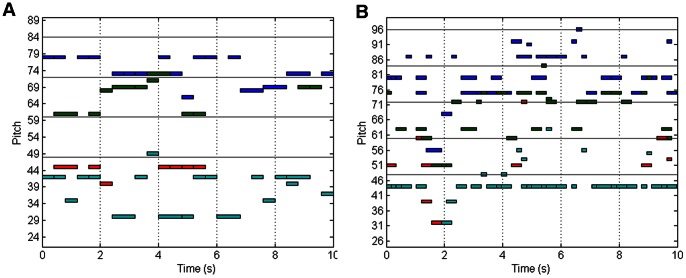
A piece of brain music from the resting EEG of a subject. Panel A showed a 10 s example of the resting brainwave quartet during eyes closed and panel B showed 10 s quartet during eyes open.

Music of the brain activities during resting states was generated for each subject. Figure 2 showed an example (Subject #5) of the quartet with eyes closed and eyes open. The audio files were provided in Supporting Information (Audio S1, Audio S2). We found that the notes in EC music were longer in duration, lower in pitch and slower in tempo, which demonstrated a peaceful and quiet mood corresponding to the EC state. In contrast, the notes of EO were shorter in duration, higher in pitch and faster in tempo, which meant that the brain was relatively alert and active.

### 2. The Comparison of Music between EC and EO Conditions

Both EC and EO music pieces were analyzed. The results indicated significant differences between the two mental states in BD and TM. The average BD of the EC manipulation was 0.29±0.09 *s*, while BD of EO was 0.24±0.08 *s* (*p*<0.05). Meanwhile, the TM of EC music (70.55±10.09 beats per minute) was slower than that of EO music (79.28±15.26 beats per minute) (*p*<0.05).

In the proposed method, the tonality filtering was a process to extract the notes belonging to a defined key. Therefore, it was obvious that the pitch distribution was changed after the filtering. We compared the scale-free exponent of pitch distribution of the EC and EO music before and after the filtering.

The number of each pitch occurring in a music piece can be counted, and then it can be sorted by a descending order. We can plot a figure according to the “Rank” and the “Number of occurrences” in logarithm coordinates. A straight line can be fitted and the slope of the line is the scale-free exponent that we want to obtain [14]. In the fitting, there is a parameter R used to represent the fitness of line and a scatter of points in the figure. The range of R is from 0 to 1. When R equals 1, the fitting is perfect; while R is near 0, the points are not fitting well by the line. In this study, R of 0.6 was the threshold for the fitting. If R<0.6, the point with the smallest occurrence would be ignored, and the line would be calculated again.

**Figure 3:**
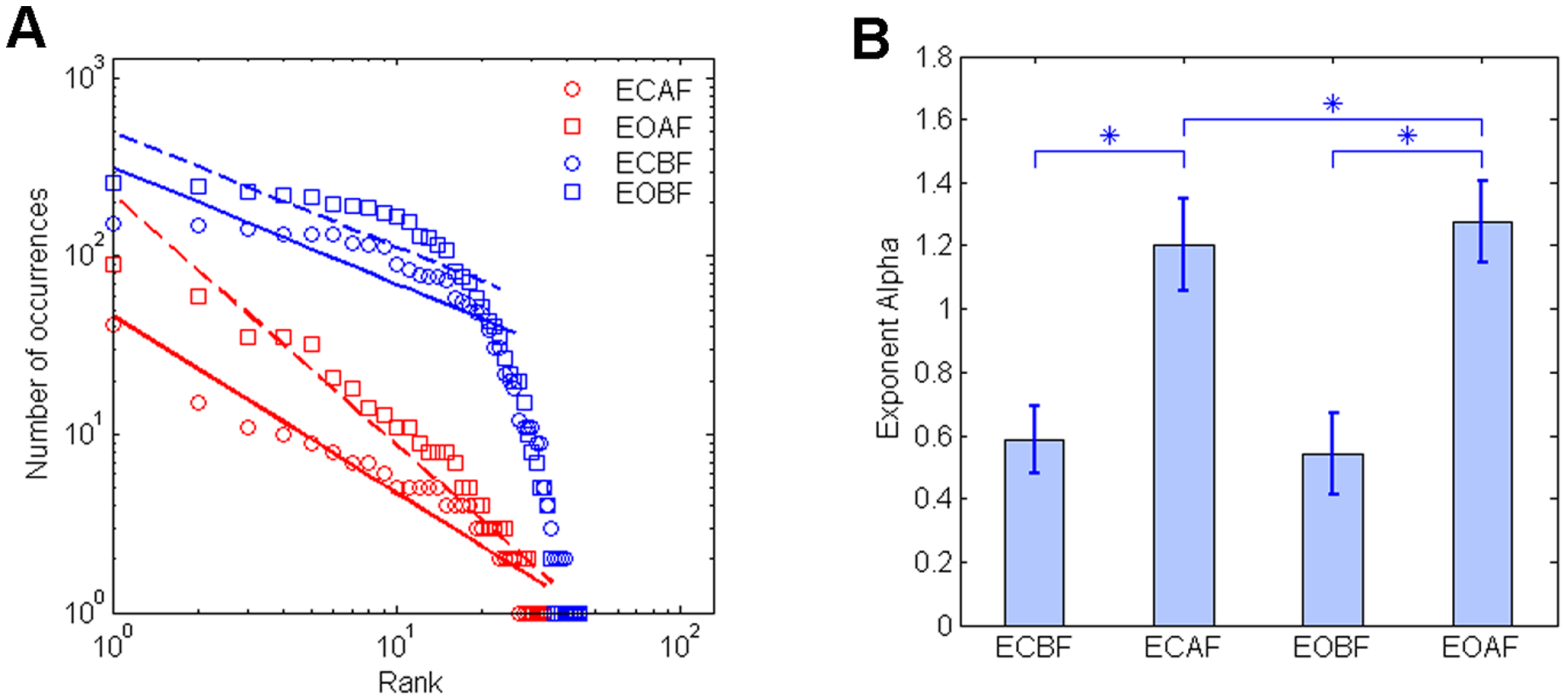
The scale-free exponents before and after the tonality filtering during rest states. There were four kinds of music pieces, including the eyes closed after filtering (ECAF), eyes open after filtering (EOAF), eyes closed before filtering (ECBF) and eyes open before filtering (EOBF).The results showed in panel A was from subject #22. Panel B showed the average for all the 40 subjects.

Figure 3A depicted the results for subject #22. There were four kinds of music: eyes closed after filtering (ECAF), eyes open after filtering (EOAF), eyes closed before filtering (ECBF) and eyes open before filtering (EOBF). We found that the distributions of ECBF and EOBF seemed like a curve, so the slope of the line was generally represented the left part of the points. The distributions of ECAF and EOAF here showed a quite good fitting of the straight line though fitting demands two lines in some cases [5].

The results of all the subjects were illustrated in Figure 3B. The average scale-free exponents of ECBF and ECAF were 0.58 and 1.2, suggesting a significant change (*p*<0.05). And the exponents of EOBF and EOAF were 0.54 and 1.27; they also changed significantly (*p*<0.05). It was more interesting that the EC and EO music pieces were significantly different after filtered (ECAF vs. EOAF), but the music could not be differentiated before filtered.

### 3. Quartet and One-channel Music

The four music pieces in the evaluation test were the single channel music with eyes closed (SEC), the single channel music with eyes open (SEO), the multi-channel music with eyes closed (MEC) and the multi-channel music with eyes open (MEO). Between MEC and MEO, there were significant differences in tempo, valence, arousal (*p*<0.05), whereas the rhythm, musicality and richness showed no differences. MEC was slower than MEO and rated lower in valence and arousal. This was in accord with the mode of the two pieces of music. MEC was Minor, which represented negative emotion, while MEO was Major, which usually was positive. There were no significant differences between the SEC and SEO in all the six parameters.

Comparing the single channel music with the multi-channel music in pairs (SEC vs. MEC, SEO vs. MEO), we found that the two types were different in tempo, rhythm, musicality and richness. The multi-channel music was slower than the single channel music because of the beat filtering and also more rhythmical, musical and rich. These results demonstrated that the quartets obtained from multiple channels were evaluated higher than the single channel music.

### 4. The Representative Regions for Different Brain States

**Figure 4:**
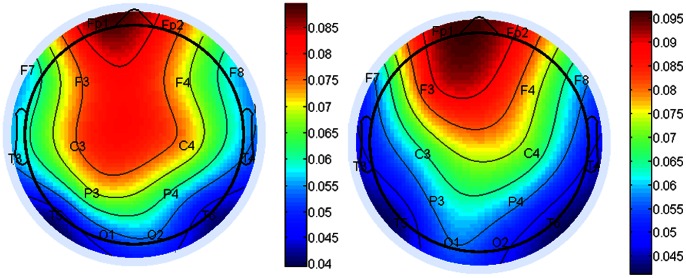
Thetopographic map of the probability of electrodes which were represented. The topographic map was the average probability for all the 40 subjects, the left was the result of brainwave music during eyes closed and the right was that during eyes open.

In fact, the tonality filtering was a method for note selection. The notes selected in every moment were generated from signals of several electrodes. Along the music sequences, we analyzed the electrodes or regions which were selected more frequently in the music generation. Figure 4 showed the probability of the electrodes selected with eyes closed (left) and eyes open (right). In both resting states, frontal electrodes were employed often. Parietal electrodes were selected more often in the EC state than in the EO state. P3 and T3 suggested larger probability in the EC state, and Fp2, F3 and F4 showed larger probability in the EO state. The results demonstrated the brain regions involved in different mental states.

## Discussion

### 1. Artistic Filter

In the proposed method, multi-channel EEGs are translated into a brain quartet, which is a new attempt to analyze the brain activities in an auditory way. Beats and tonality filters are used to expand the single channel brain music into a quartet. In fact, there are many kinds of translating methods. The selecting criterion is corresponding to the aim. For the research which aimed at to monitor the features of EEG, the method sensitive to the waveforms was chosen [6]; when the entertainment was concerned, some computer music technologies were used [8]. In this work, we tried to listen to the brain activities in a relatively objective way, so the scale-free method was chosen. On the other hand, we also wanted to represent the brain in a musical way; thus an artistic filter was designed for music generation. Such a filter is designed based on the assumption that musicians adopt artistic filters to turn natural materials into real music. The filtered brain music is scale-free, and the exponent is closer to 1. It is by no accident. Signals from nature or body are scale-free in fact, and filters of the artists obey, even enhance, the properties of the signals. Certainly, as physiological signals including EEGs are scale-free, it is reasonable that a quartet generated by the brain activities has the similar scale exponent with some classical music works.

EEG data are recorded from electrodes on different regions of the brain. How to represent the important temporal and spatial features in the brain music or audios is the core of a music generation strategy. If we simply put together the melodies from each channel [15], it may result in a cacophony of dissonant sounds which are hard to identify. The proposed method uses the filter which is common in signal processing to imitate the artists’ composing. In music, a quartet is an ensemble of four singers or instrumental performers, or a musical composition for four voices or instruments [16]. In a broader sense, a music having four parts can be perceived as a quartet, where the four parts are organized, performed together or solo, to express the theme of the work. Each part has its own melody, but the organization of all the parts based on the harmony rules makes a whole composition. Actually, the brain may work in a similar way; that is, there are various combinative patterns of brain regions during the processes of different functions, and whether a special region is involved or not is up to the function. Therefore, we believe that an artistic filter can help us to understand the brain. Results of the evaluation test in the current work showed the multi-channel music was more rhythmical and more musical than the single channel music.

### 2. Tonality Filter

The tonality filtering extracts notes from four out of multiple channels and makes the music from atonal to tonal. The definition of music key is a strategy for finding the most important information of electrodes from a musical aspect. The music tonality is a theory of relations between the notes in music pieces, and in these systems, a topic, which may express certain meanings or emotions, can be represented. So the tonality filtering is a way to extract the information in the signals from all electrodes. The filtering selects the amplitude which occurs most frequently because the pitch in the brain music is based on the amplitude of the EEG.

On the other hand, the music has a key after filtering. The selection of notes is based on the tonal stability. Some studies have revealed that scale-free exponents of the parameters of music may influence aesthetics, when the exponent is near 1, music is regarded as “just right”; and when the exponent is approaching 0, music becomes “too random”; when the exponent is near 2, music is “too correlated” [14], [17]. If a music piece is atonal, the exponent of the pitch distribution may be near 0, and the exponent of a tonal music piece is probably around 1 because the pitches are arranged in hierarchies [14], [18]. The comparison of the music in two mental states in the present research revealed that the scale-free exponent changed after filtering and became closer to 1. So the resulted sequences can be considered as a musical extraction of EEG signals.

### 3. Multi-channel Music

Multi-channel music can be understood easily. Differences of brain states are identified by the intrinsic characters of music, such as pitch, duration and tempo. In this study, EC music was longer in duration and slower in tempo than EO music. This is consistent with the features of the two mental states; eyes-closed state usually means peace and quiet whereas the brain activities are often increased with eyes open.

Compared with the single channel music, the quartet was tonal, which reflects the pitch hierarchy; the quartet was more rhythmical and in line with the brainwave frequency: the durations of notes were more regular and repetitive. The volume of notes was determined by the power of the alpha band, which revealed the main amplitude of the brain activities. Besides, if there is a synchronous fMRI recording, the amplitude of the BLOD signals may be utilized to control the intensity [9]. In general, the brain quartet was developed in pitch, duration and intensity, so it sounded more musical and interesting.

As we know, individual differences existed in the EEG signals, so the brain quartets of different subjects were not exactly the same in the same condition. However, the statistic results indicated that the varieties between mental conditions were relatively stable though the music for the subjects might be of different pitch, tempo and tonality (Figure 3B).

The analysis of the relative regions showed that during the EO state, signals from frontal areas were included more than those from other regions, and during EC state, parietal information was employed more often. Generally speaking, frontal signals are of high frequency and low amplitude (beta bands for example), which means high stability. That may be the reason why frontal areas showed higher probability of selection than other regions.

### Conclusion

In conclusion, we discovered a method for translating multi-channel EEGs to a quartet based on the artistic filters. The artistic filters are the imitation of artists’ composition, and such imitation is based on the scale-free properties. In general, musicians have been inspired by the nature and they have abstracted information into their compositions. The scale-free, or say the power law, exists in the natural phenomena and music. Our artistic filters also substantiate the power law in brain music. Furthermore, the proposed method may provide a more sensitive way to detect subtle variations of EEG, which might be ignored by conventional EEG waveform techniques. The properties of scale-free are important for signal transferring and processing [19], [20], so filtering the scale-free pitches in the brain activities is a worthwhile attempt, and it may be used as a new approach for EEG analyses and applications.

## Supporting Information

Audio S1 **60 s brain music with eyes closed.**
(MP3)

Audio S2 **60 s brain music with eyes open.**
(MP3)
